# Implications of morphological and molecular distinctness on the registration of alfalfa candidate varieties issued by a breeding program: a case study

**DOI:** 10.3389/fpls.2026.1809996

**Published:** 2026-04-14

**Authors:** Paolo Annicchiarico, Nicolò Franguelli, Luciano Pecetti, Barbara Ferrari, Nelson Nazzicari

**Affiliations:** Research Centre for Animal Production and Aquaculture, Council for Agricultural Research and Economics (CREA), Lodi, Italy

**Keywords:** DUS testing, genetic diversity, *Medicago sativa*, molecular marker, morphological trait, plant variety protection, variety discrimination, variety registration

## Abstract

Candidate varieties must satisfy the requirements of Distinctness, Uniformity and Stability (DUS) based on morphophysiological traits. Breeders of major perennial forage crops strive to achieve statistically significant distinctness of candidate varieties, especially with respect to registered varieties bred from the same genetic base. This study aimed to compare morphophysiological *versus* molecular distinctness for 10 potential alfalfa varieties selected from the same genetic base and one recently registered variety selected from a similar genetic base. We also aimed to support the optimization of alfalfa molecular distinctness by comparing (a) genotyping-by-sequencing (GBS) *versus* alfalfa DArTag panel markers, and (b) four statistical criteria. The 11 populations (one originating from mass selection, nine from progeny-based selection, and one from clonal selection) were evaluated for 10 morphophysiological traits ordinarily used for DUS in Italy and were characterized molecularly using three bulked DNA samples of 200 independent plants each per population. Only four morphophysiological traits exhibited significant (*P* < 0.01) population variation, mainly because of population similarity for autumn dormancy, growth habit, and plant vigor. Morphophysiological distinctness emerged for 20 of the 55 paired comparisons between the 11 populations, with no population showing distinctness from any other. Various GBS and DArTag marker configurations achieved a complete distinctness of each population from any other (at *P* < 0.01) by statistical criteria based on a principal components analysis of allele frequencies followed by analysis of variance or discriminant analysis of population principal component scores. These criteria showed greater population discrimination than cluster analysis with bootstrap values. Analysis of molecular variance was ineffective for population distinctness, probably because of insufficient number of bulked DNA samples per population. Mantel’s test indicated high correlation for Euclidean distances of the populations between GBS and DArTag markers (*r* = 0.94) and no correlation of these distances with those based on morphophysiological traits. Variety registration on the ground of distinctness from the already registered variety could be granted to only four of 10 selections according to morphophysiological distinctness, and to all selections according to molecular distinctness. Our results support molecular distinctness as a sensitive, quick, and inexpensive tool for alfalfa variety registration, forensic analyses, and control of certified seed.

## Introduction

Registration and protection of plant varieties are usually carried out according to regulations set by the International Union for the Protection of New Varieties of Plants (UPOV), an international organization whose members cover 99 states. To be registered, a candidate variety must satisfy the requirements of Distinctness, Uniformity, and Stability (DUS) (UPOV, 1991). Variety distinctness, which is the main requirement, is also important for authorities that control and certify seed production chains, and in lawsuits regarding illicit seed marketing, variety plagiarism, and essential derivation claims (Van Wijk and Louwaars, 2014). According to UPOV (1991), a variety “shall be deemed to be distinct if it is clearly distinguishable from any other variety whose existence is a matter of common knowledge at the time of the filing of the application”. The distinctness requirement is satisfied if at least one statistically significant difference can be detected between the candidate variety and each registered cultivar in the DUS assessment across a set of species-specific morphophysiological characteristics that do not need to be of agronomic value (UPOV, 2015). In the EU, where registration in the national list of one member state is mandatory for variety marketing, candidate varieties must also satisfy the requirement of sufficiently high Value for Cultivation and Use (VCU).

The requirement of variety distinctness, which was conceived in a period featuring fairly small numbers and long commercial lifespan of the registered varieties, is bound to be increasingly difficult to satisfy for candidate varieties of species including a large number of registered varieties. This complication is enhanced, in outbred species bred as a synthetic variety such as the perennial forages, by the difficulty of detecting statistically significant differences due to substantial intra-variety morphophysiological variation. This variation, which reportedly exceeded the size of inter-variety variation for many traits (Annicchiarico et al., 2015; Herrmann et al., 2018), is caused by the need to select at least 7–8 parent plants for a synthetic variety to limit the risk of inbreeding depression (Busbice, 1969). An earlier study revealed that the vast majority of the 18 most grown Italian commercial varieties of alfalfa (*Medicago sativa* L. subsp. *sativa*) was not completely distinct according to conventional DUS based on morphophysiological traits (Annicchiarico et al., 2025). The successful registration of these varieties was favored by the fact that the DUS requirements of a candidate variety are actually verified with respect to a small number of registered varieties (which are four for alfalfa in Italy), to limit the variety registration costs. However, even a limited assessment of distinctness does not prevent the rejection of many agronomically valuable candidate varieties due to insufficient distinctness. Twelve out of 46 candidate varieties proposed in France over the period 2001–2010 were rejected essentially because of insufficient distinctness while featuring sufficient VCU (V. Gensollen, M.-C. Gras and B. Julier, pers. comm., 2016). Over 12% of the candidate varieties of perennial ryegrass (*Lolium perenne* L.) or white clover (*Trifolium repens* L.) were rejected for insufficient distinctness in the UK over the period 2000-2008 (Gilliland and Gensollen, 2010). The number of rejected candidate varieties would be much higher if their distinctness was tested with respect to all the registered varieties. For alfalfa, the EU database reports 498 currently registered varieties (EUPVP, 2025), while over one thousand cultivars are listed in the OECD catalogue (OECD, 2025). Italy provided a sizeable share of the alfalfa varieties registered in the EU, namely, about 30% (EUPVP, 2025). Additional limitations of the morphophysiological trait-based distinctness are represented by the duration and high cost of its assessment, which requires the observation of a number of traits on many plants under field conditions over at least two years. These limitations complicate its application also for forensic studies or the timely control of seed production chains (Gilliland and Gensollen, 2010; Jamali et al., 2019, 2020).

The limitations featuring the current assessment of forage crop variety distinctness prompted several scientists to envisage and verify the value of molecular marker-based variety distinctness (Roldán-Ruiz et al., 2001; Byrne et al., 2013; Yu et al., 2022; Annicchiarico et al., 2016b, 2025; Julier et al., 2018). The exploitation of molecular distinctness in the absence of morphophysiological distinctness is a cornerstone of a revised variety registration procedure for forage crops proposed by Gilliland et al. (2020). They recommended to verify the molecular distinctness on the basis of the overall diversity across a large number of single nucleotide polymorphism (SNP) markers rather than based on differences for a few markers as envisaged in early studies (e.g., Pupilli et al., 1996) to minimize the risk of registration of essentially-derived varieties selected by a drastic change of allele frequencies for a few markers – which is a major concern associated to the use of molecular distinctness (Gilliland and Gensollen, 2010; UPOV, 2004). The molecular assessment could be performed using a few independent bulked DNA samples per cultivar (each pooling 100–200 individual plants and acting as an experiment replicate). This sampling strategy has several advantages over the analysis of a large number of individual plants per cultivar, namely, a much lower evaluation cost, a broader plant population sampling, and a possible reduction of sampling bias arising from rare marker alleles (Pupilli et al., 1996), while providing similar results (Julier et al., 2018). Even molecular distinctness, however, has to face the difficulty represented by greater diversity within cultivars than between cultivars (Herrmann et al., 2018; Malmberg et al., 2025; Wang et al., 2025). The adoption of molecular distinctness has also been proposed for a non-forage outbred crop such as cabbage (Jo et al., 2022), as well as for major inbred crops, for which the morphophysiological trait-based distinctness is less challenging but is limited by its high costs and increasing difficulties of application to very large variety numbers (Owen et al., 2019; Achard et al., 2020; Yang et al., 2021).

The statistical assessment of molecular distinctness, which should be based on Type 1 error rates just like morphophysiological distinctness, and the optimal marker panel, are important aspects for the implementation of marker-based distinctness procedures. Granting distinctness according to a minimal threshold of genetic distance, as investigated mainly for inbred crops (Van Eeuwijk and Law, 2004; Jones et al., 2013), requires the prior determination of a reliable, crop-specific threshold through extensive research work. Yu et al. (2022) considered a set of ryegrass varieties to be molecularly distinct if a hierarchical cluster analysis with bootstrap values was able to separate the bulked DNA samples (i.e., the replicates) of each cultivar into a distinct group. Julier et al. (2018) envisaged the paired comparisons of alfalfa varieties by an analysis of molecular variance (AMOVA). Annicchiarico et al. (2016b) envisaged two methods for assessing the molecular distinctness of alfalfa cultivars that involved an initial principal components analysis (PCA) of the bulked DNA samples aimed at dimensionality reduction of the overall variation in marker allele frequencies. One method envisaged paired cultivar comparisons by an analysis of variance (ANOVA) of the cultivar principal component (PC) scores for each PC axis (considered as a distinct variable); the other method was based on a discriminant analysis of the cultivars based on their PC score values. An empirical comparison of all these statistical procedures based on their ability to distinguish alfalfa varieties revealed the high interest of PCA + ANOVA and the good performance of PCA + discriminant analysis and cluster analysis, while AMOVA was strongly limited by the fairly low number of bulked DNA replicates (which were three per cultivar) (Annicchiarico et al., 2025). Two possible marker types have been proposed for alfalfa distinctness, namely, those issued from genotyping-by-sequencing (GBS) (Annicchiarico et al., 2016b; Julier et al., 2018), and the recently developed open-access alfalfa DArTag panel of 3,000 loci (Zhao et al., 2023). The latter, featuring lower genotyping fees and somewhat easier data management (e.g., in terms of missing data) albeit at the cost of lower marker number in comparison with GBS, exhibited a slight advantage in terms of cultivar distinctness in an empirical comparison (Annicchiarico et al., 2025).

The implementation of molecular distinctness in DUS testing of major forage crops depends crucially on the scientific evidence for greater sensitivity than morphophysiological distinctness. Two earlier studies on alfalfa, one based on GBS markers for distinction of Italian historical commercial ecotypes (Annicchiarico et al., 2016b) and the other envisaging GBS or DArTag markers for distinction of the 18 most grown Italian commercial varieties (Annicchiarico et al., 2025), revealed a definite increase in the number of distinct cultivars obtained by molecular distinctness based on PCA + ANOVA compared with morphophysiological distinctness. In addition, they indicated a limited increase of distinct cultivars when combining molecular and morphophysiological distinctness in comparison with molecular distinctness alone despite the fairly modest correlation between molecular and morphophysiological genetic distances, owing to the high sensitivity of the molecular criterion. However, neither of these studies fully represent the implications of introducing a marker-based distinctness assessment from a breeder’s viewpoint. Because of the widespread adoption of recurrent selection within restricted breeding populations (Wilkins and Humphreys, 2003), a key challenge for forage crop breeders is achieving sufficient distinctness of own candidate varieties relative to earlier registered varieties of theirs that derived from the same or a closely related genetic base. Selecting various candidate varieties from the same genetic base may increase in the future because of the introduction of genomic selection schemes by which a genomic prediction model is applied repeatedly on the same reference population (Li and Brummer, 2012).

The main objective of this study was to compare morphophysiological *versus* molecular distinctness on 10 potential alfalfa varieties and one recently registered variety that were selected from the same or a similar genetic base. In addition, our study aimed to contribute to the optimization of molecular distinctness in alfalfa by comparing (a) GBS *versus* DArTag panel markers and (b) four statistical criteria for variety distinctness, as well as verifying the consistency of information on cultivar diversity provided by morphophysiological and molecular data.

## Materials and methods

### Plant material

Our study was based on morphophysiological and molecular data of 11 selections of alfalfa (termed populations hereafter) that were bred in Northern Italy from genetic bases formed by elite genotypes issued from prior stages of mass selection performed on landrace and variety germplasm from the Po Valley. One of them was the recently registered synthetic variety ‘Intensa’, which corresponded to the coded selection GSA in Annicchiarico (2021) and included 15 parent genotypes selected for specific adaptation to one subregion of Northern Italy from 90 elite parent genotypes (**Table 1**). The remaining 10 populations were experimental synthetic varieties originating from a genetic base similar to that of ‘Intensa’, which are thoroughly described in Annicchiarico and Pecetti (2021). Eight of them included 12 S_0_ or S_1_ parent genotypes (S_0_ and S_1_ = derived from plants that were not selfed and selfed for one generation, respectively) that were selected from an initial genetic base of 125 mass-selected genotypes by some type of progeny testing, as frequently performed in forage crop breeding (Pohelman and Sleper, 1995; Wilkins and Humphreys, 2003; Annicchiarico et al., 2016a); one derived from clonal selection of 12 S_0_ parents out of the same 125 mass-selected genotypes; and one included all the 125 mass-selected genotypes as parents (**Table 1**). The 11 populations were coded by alphabetic letters as reported in **Table 1** for display of results.

**Table 1:** Code and description of 11 alfalfa populations selected from the same or a similar genetic base of Po Valley germplasm.

Entry code	Genetic base^a^	Description of the selected material
A	1	Entire genetic base of 125 mass-selected parents^b^
B	1	Selection scheme 1: clonal selection of 12 S_0_ parents^b^
C	1	Selection scheme 2: half-sib progeny-based selection of 12 S_0_ parents^b^
D	1	Selection scheme 3: half-sib progeny + within-progeny selection of 12 S_0_ parents^b^
E	1	Selection scheme 4: S_1_ progeny-based selection of 12 S_0_ parents^b^
F	1	Selection scheme 5: S_1_ progeny + within-progeny selection of 12 S_1_ parents^b^
G	1	Selection scheme 6: S_2_ progeny-based selection of 12 S_0_ parents^b^
H	1	Selection scheme 7: S_2_ progeny-based + within-progeny selection of 12 S_1_ parents^b^
J	1	Selection scheme 8: S_2_ progeny-based selection of 1 S_1_ parent for each of 12 best S_0_ parents^b^
K	1	Selection scheme 9: S_2_ progeny-based selection of 12 S_1_ parents across all S_1_ genotypes^b^
M	2	Half-sib progeny-based selection of 15 S_0_ parents for specific adaptation to subregion A^c^

^a^ Genetic base 1 and 2: 125 and 90 possible parent genotypes, respectively, issued from an initial stage of mass selection.

^b^ See Annicchiarico and Pecetti (2021) for details. S_0_ and S_1_ = derived from plants that were not selfed and selfed for one generation, respectively.

^c^ See Annicchiarico (2021) for details (where the population is coded as GSA). Registered in Italy in 2025 as ‘Intensa’.

### Morphophysiological data

The morphophysiological characterization of the 11 populations was performed in Lodi, Northern Italy (45°19′ N, 9°30′, 81 m a.s.l.), by an experiment laid out as a randomized complete block with five replications. Seeds were sown in polystyrene plug-trays in early September 2015. The seedlings were transplanted six weeks later in a field that was previously fertilized with 100 kg/ha of N, 50 kg/ha of P_2_O_5_, and 200 kg/ha of K_2_O. Moderately favorable growing conditions were ensured by providing 220 mm of irrigation water distributed into four summer irrigations. The following 10 traits were recorded during 2016 on 22 plants per plot (excluding plot edge plants, acting as borders): (a) plant height in spring (one month after the beginning of growth); (b) onset of flowering in spring measured on the regrowth after the first harvest (as number of days from June 1); proportions of plants with (c) very dark blue-violet flower, (d) variegated flower, and (e) cream, white or yellow flowers, all observed in spring; (f) length of the tallest stem at full flowering stage, recorded in late spring; (g) length and (h) width of the central leaflet, measured on the fourth leaf below the inflorescence of the tallest stem in late spring; (i) plant growth habit, assessed two weeks before the autumn equinox on a scale ranging from 1 = erect to 9 = prostrate; (j) plant height in autumn, two weeks after the autumn equinox. Our DUS assessment reflected that performed in Italy and was largely consistent with UPOV’s recommendations regarding mandatory traits and observation procedures for alfalfa variety registration trials (UPOV, 2005). We included all mandatory traits and added three non-mandatory traits, namely, plant growth habit, and length and width of the central leaflet, to increase the cultivar discriminating ability of the assessment. Compared with DUS trials in Italy, which adopt four-replicate experiments and 25 measured plants per plot, our experiment included one extra replicate and a somewhat larger total number of measured plants per population (110 instead of 100). Another difference was represented by the adoption of one evaluation trial, instead of two trials sown in subsequent years.

### Molecular marker data

The molecular characterization was identical to that reported in Annicchiarico et al. (2025) for commercial cultivars of alfalfa. Plant sampling for DNA analyses was performed on young plants grown in a greenhouse. Each population was genotyped using three bulks of 200 independent plants each, obtained by pooling the central leaflet from the first trifoliate leaf of each plant. Genomic DNA was extracted from each bulked DNA sample using the DNeasy Plant Mini Kit (Qiagen, Milan, Italy). Nucleic acid was quantified by a Quant-iT™ PicoGreen™ dsDNA Assay Kit (P7589, Life Technologies, Milan, Italy), checking its quality by 1% agarose gel electrophoresis. A trial digestion process was carried out on 10% of the 33 DNA samples using the Optizyme EcoRI restriction enzyme (25,000 U, Fisher BioReagents, Rodano, MI, Italy), to compare bands of cut and uncut DNA.

The GBS-based characterization of the populations was carried out by the Elshire Group Ltd. laboratory (Palmerston North, New Zealand) according to the method described by Elshire et al. (2011), restricting the genomic DNA by the ApeKI enzyme (NEB New England Biolabs, Ipswich, MA, USA, R0643L). Modifications to the method concerned the use of 100 ng of genomic DNA and 3.6 ng of total adapters, and the library amplification by Kapa Taq polymerase α (KAPA Library Amplification Readymix, Kapa Biosystems, Wilmington, MA, USA, KK2611) through 18 PCR cycles. The library was sequenced using an Illumina X Ten platform with 150 bp paired end reads. After demultiplexing, SNP data were aligned along the reference genome published by Long et al. (2022). The SNP calling was then performed using the Legpipe2 pipeline described by Nazzicari et al. (2024).

The DArTag marker-based characterization of the 33 bulked DNA samples was performed by the Diversity Array Technology Pty Ltd of the University of Canberra, Bruce (Australia) according to the Alfalfa_DArTag_BI_Cornell_University (1.0) tool developed in collaboration by DArT and Breeding Insight at Cornell University (Zhao et al., 2023). Sequencing was performed on Illumina Hiseq2500/Novaseq6000. We received allele counts of the SNP markers aligned along the reference genome, after the outsourced SNP calling performed through the DArT P/L’s proprietary pipeline (Diversity Arrays Technology, 2026).

Molecular marker data (either from GBS or DArTag) were represented as SNP allele frequencies for each relevant marker. Several filtering configurations were tested to optimize the power of distinctness analyses. The markers were filtered for minor allele frequency greater than 5% (computed as average frequency over all samples), as well as for different filtering scenarios relative to minimum number of reads per locus (10, 20, 30 or 40) and maximum allowed missing rate (1%, 5%, 10%, 20%, 30%) which resulted, overall, in 20 different filtering configurations of genotyping data for each genotyping method (**Supplementary Table 1**). Missing data points that were retained after filtering were imputed via Random Forest Imputation on the continuous domain.

### Statistical analysis

Morphophysiological traits observed on individual plants of each plot (growth habit, onset of flowering, plant height, stem length, length and width of the central leaflet) were previously averaged to obtain a plot value used for statistical analyses. For plant growth habit, the populations were considered to differ when their difference exceeded 1.5 units, according to UPOV’s (1990) indications for the analysis of quantitative traits observed on a discrete scale. The occurrence of statistical differences for the other morphophysiological trait was assessed by ANOVA and, in the presence of overall population variation at *P* < 0.05, by pairwise comparisons based on Fisher’s least significant difference at *P* < 0.01, according to UPOV’s (2022) recommendations for quantitative traits featuring continuous variation. The ANOVA was performed on data submitted to the angular transformation for frequencies of flower colors. Onset of flowering was analyzed as a quantitative trait since the distribution of its residuals from ANOVA tended to be normal according to the Shapiro-Wilk test. After performing the 55 possible pairwise population comparisons for each trait, we computed the proportion of comparisons that exhibited no statistical difference for any trait (i.e., the undistinguished pairs of populations) and those that showed one or more trait differences. Likewise, we verified the number of completely distinct populations, i.e., the populations that differed from any of the other populations for at least one trait in the pairwise comparisons. Phenotypic correlations between traits of the 11 populations were also assessed.

The occurrence of molecular marker-based statistical differences for each of the 55 possible paired population comparisons was assessed separately for each genotyping method (GBS or DArTag) by different statistical criteria as performed in a prior study on alfalfa cultivars (Annicchiarico et al., 2025). One criterion involved the performance of a PCA on marker data of the 33 bulked DNA samples, followed by a separate one-way ANOVA for each PC axis performed on the population PC scores in which the bulked DNA samples acted as replicates of the populations. Consistently with results for morphophysiological traits, two populations were considered distinct if they differed at *P* < 0.01 in at least one ANOVA performed on population PC score values. The first ANOVA was performed on data of PC 1, marking as distinct all the pairs that displayed significantly different PC scores. Subsequent ANOVAs progressively assessed the occurrence of significant differences for the other pairs of populations for higher-ranking PC axes, until no population difference was observed for three subsequent PC axes. The normal distribution of population PC scores was verified by the Shapiro-Wilk test. The set of ANOVAs which defined the distinct or non-distinct paired population comparisons was repeated for each of the different combinations of filtering levels available for each genotyping method. The ability to distinguish populations was assessed not only on the basis of the paired comparisons but also according to the number of completely distinct populations (i.e., those that exhibited distinctness in any pairwise population comparison).

A second criterion for assessing the occurrence of molecular distinctness between populations relied on a linear discriminant analysis. For each of the 55 paired population comparison, we performed a separate PCA aimed at dimensionality reduction on the bulked DNA samples, retaining the first five PC axes (given the six observations per comparison). Then, we performed a discriminant analysis in a leave-one-out scheme: five samples were used to tune the model, and one was predicted and compared with the population to which the sample belonged. The analysis was repeated, in turn, for each of the six samples, and two populations were considered distinct if the model was able to correctly classify all of them. The analysis was performed considering an increasing number of PC axes from one to five, and was repeated for all the filtering configurations, retaining the most-discriminating configuration for each genotyping method. Also here, we computed the number of distinct populations in paired population comparisons and that of completely distinct populations.

A third criterion for assessing marker-based cultivar differences relied on the analysis of molecular variance (AMOVA) as proposed by Julier et al. (2018). For each combination of filtering level and genotyping method, Nei’s (1972) distance matrix between pairs of populations was computed, and the AMOVA for each population pair was performed using the function “stamppAmova” of the StAMPP R package version 1.6.3 (Pembleton et al., 2013) with the parameter “nperm = 10000”. In this case, two populations were considered distinct if they differed at *P* < 0.05 in the AMOVA. The current adoption of a more liberal *P* value was justified by the fact that cultivar distinctness was based on a single statistical test, rather than several tests as in the case of several morphophysiological traits or PC axes (whose overall cultivar distinctness result implied a cumulation of *P* < 0.01 error rates). For this criterion, the following one, and Mantel’s test, the analysis was performed on data of a filtering configuration that proved well-discriminating in earlier analyses.

One last criterion for assessing marker-based population distinctness was inspired by the criterion in Yu et al. (2022) based on hierarchical cluster analysis modified as described in Annicchiarico et al. (2025). One population was distinct from any other population if its bulked DNA samples (replicates) were classified unequivocally and exclusively in the same group according to a probability threshold for group membership (shown at the node of the relevant group) set by bootstrap analysis within a hierarchical cluster analysis. We built the phylogenetic tree by the UPGMA (unweighted pair group method with arithmetic mean) method (Khan et al., 2008; Vaz et al., 2021) in a cluster analysis performed on the correlation-based distance matrix constructed from allele frequency data. The cluster analysis was repeated via bootstrapping with 1000 replications, estimating approximate probability values according to Shimodaira (2002). This procedure was implemented using the pvclust R package (Suzuki et al., 2019). We constructed consensus phylogenic trees for GBS and DArTag markers and adopted a probability threshold ≥ 95% (analogous to a *P* < 0.05 error rate) for the exclusive classification of all population replicates in the same group. Because of the absence of explicit pairwise comparisons, the results of this analysis were expressed only in terms of number of populations that were distinct from any other population.

The consistency of cultivar diversity information provided by morphophysiological traits showing significant (*P* < 0.05) population variation, GBS markers, and DArTag markers was verified by assessing the correlations between Euclidean distance matrices computed for each information layer by Mantel’s test using the mantel() function of the R package “vegan” (Legendre and Legendre, 2012), averaging the results over 10,000 permutations.

## Results

### Morphophysiological trait-based distinctness

Only four traits exhibited significant (*P* < 0.05) variation among populations, namely, frequency of plants with variegated flowers, which showed the highest ability to distinguish populations (12 out of 55 in paired comparisons), onset of flowering (five distinct paired comparisons), frequency of plants with very dark flower (four distinct paired comparisons), and length of central leaflet (two distinct paired comparisons) (**Table 2**). These traits showed no correlation (*P* > 0.10). No significant variation was observed for plant height in spring or autumn (indicative of the extent of autumn dormancy), length of the tallest stem at flowering, and plant growth habit, indicating that the selections had similar autumn dormancy and plant vigor and a consistently erect growth habit. No single plant showed any cream, white, or yellow flower (**Table 2**).

**Table 2:** Population range value, overall trait significance, and number and proportion of pairs of distinct populations (*P* < 0.01) in 55 paired comparisons among 11 alfalfa populations, for 10 morphophysiological traits.

Trait	Range	Significance^a^	Number^b^	Proportion (%)
Plant height in spring (cm)	31.2 – 34.9	NS	0	0.0
Onset of flowering (no. of days from June 1)	11.3 – 14.4	*	5	9.1
Proportion of plants with very dark flower	0.00 – 0.08	*	4	7.3
Proportion of plants with variegated flower	0.04 – 0.44	**	12	21.8
Proportion of plants with cream, white or yellow flowers	0.00 – 0.00	NS	0	0.0
Length of tallest stem at full flowering (cm)	85.0 – 93.4	NS	0	0.0
Length of central leaflet (mm)	30.2 – 33.1	*	2	3.6
Width of central leaflet (mm)	11.2 – 13.7	NS	0	0.0
Plant growth habit (scale 1–9)	1.0 – 2.0	NS	0	0.0
Plant height in autumn (2 weeks after equinox; cm)	27.1 – 29.9	NS	0	0.0

^a^ NS, not significant; *, **, significant at *P* < 0.05 and *P* < 0.01, respectively. Based on the occurrence of population differences exceeding 1.5 for plant growth habit and on analysis of variance for the other traits.

^b^ Overall, 20 paired population comparisons showed distinctness for at least one trait.

Thirty-five out of 55 (i.e., 63.6%) paired comparisons showed no distinctness according to any trait; 17 comparisons showed distinctness according to one trait; and three comparisons showed distinctness based on two traits (**Supplementary Table 2**). According to expectations, the material with largest intra-population variation, i.e., the mass-selected population coded as ‘A’ (**Table 1**), showed the lowest distinctness, being distinguishable only from one other population (**Supplementary Table 2**). Even the population bred from a partly different genetic base, coded as ‘M’, was distinct only from four other populations (**Supplementary Table 2**). Importantly, no population exhibited complete distinctness, i.e., could be distinguished from any of the other 10 populations by at least one trait.

### Molecular marker-based distinctness

The numbers of polymorphic markers available for GBS and DArTag characterizations are reported in **Supplementary Table 1** for the 20 configurations identified by different thresholds of maximum missing genotypes and minimum number of reads per marker. Such numbers ranged from 1471 to over 33,500 for GBS markers, and from 1210 to 1799 for DArTag markers, passing from the most stringent configuration (a maximum of 1% missing genotypes per marker; a minimum of 40 reads per marker) to the most liberal one (a maximum of 30% missing genotypes per marker; a minimum of 10 reads per marker).

**Figure 1:**
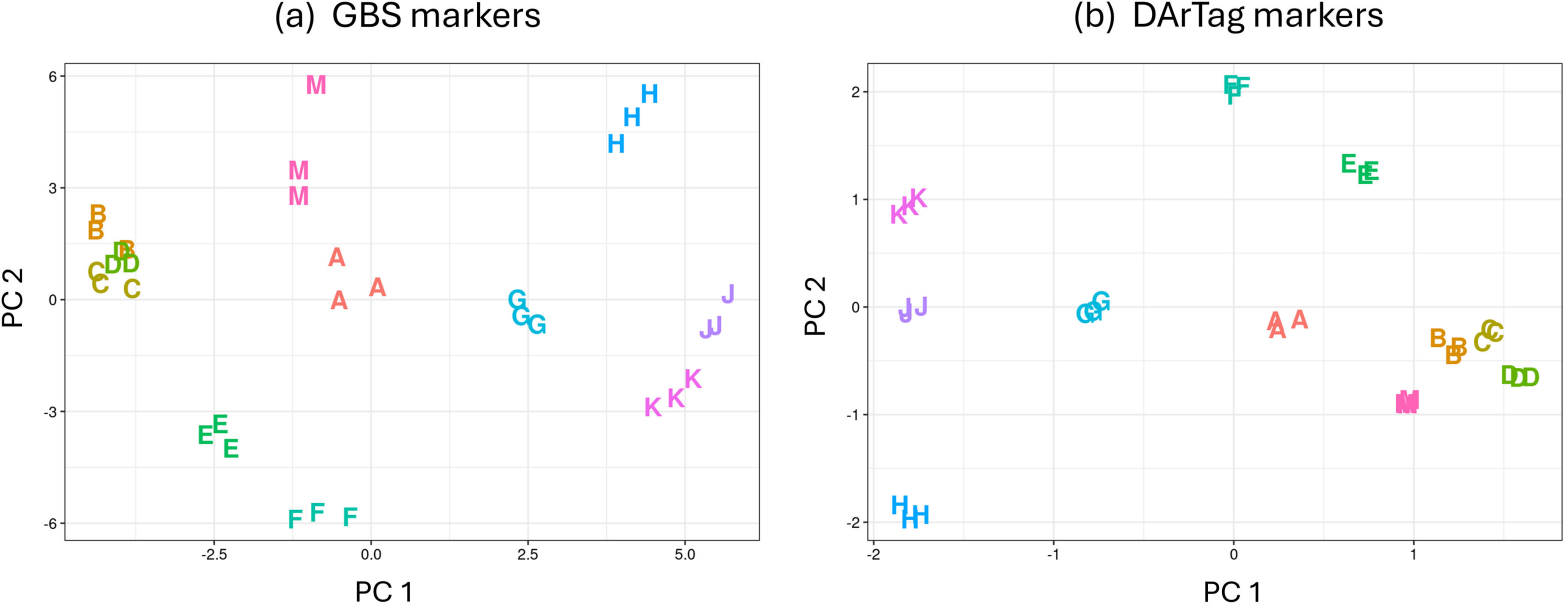
Ordination of bulked DNA samples of 11 coded alfalfa populations (three samples per population) along the first two axes (PC 1 and PC 2) of a principal components analysis performed on **(a)** 10,268 GBS-generated SNP markers, and **(b)** 1569 DArTag SNP markers.

The statistical criterion based on ANOVAs performed on population PC scores achieved a complete distinctness of all populations for both marker types (**Table 3**) and by any of the 20 possible marker configurations available for each marker type. This result was achieved by ANOVAs on population scores performed on the first 13 to 16 PC axes depending on the marker configuration for GBS, and always by ANOVAs on the first 13 PC axes for DArTag markers. A fairly good separation of the 11 populations, and similar values of the three bulked DNA samples acting as replicates of each population in the ANOVAs performed on PC scores, were already manifest for both marker types when considering the population scores on the first two PCs, as shown in **Figure 1** for a marker configuration with a maximum of 10% missing genotypes per marker and minimum of 30 reads per marker. This configuration, which included 10,268 markers for GBS and 1569 markers for DArTag, was rather stringent for requested quality of information and well-discriminating for both marker types according to the current criterion and the other one based on discriminant analysis. Therefore, it was also used for Mantel’s test and for distinctness assessments based on AMOVA or cluster analysis. The mass-selected population ‘A’ had a central position in the ordination as a function of the first two PC axes (**Figure 1**), as expected from the fact that it originated from any possible candidate parent used (as such or after selfing) for selections from ‘B’ to ‘K’ (**Table 1**).

**Table 3:** Number and proportion of (a) pairs of alfalfa distinct populations in 55 paired comparisons among 11 populations and (b) populations distinct from any other population, according to different statistical criteria.

	Distinct in paired comparisons		Distinct from any other	
Criterion	Number	Proportion (%)	Number	Proportion (%)
Morphophysiological trait-based distinctness^a^	20	36.4	0	0.0
GBS-generated SNPs, LSD in ANOVA for PC axes^b^	55	100.0	11	100.0
DArTag SNPs, LSD in ANOVA for PC axes^b^	55	100.0	11	100.0
GBS-generated SNPs, PCA followed by discriminant analysis^c^	55	100.0	11	100.0
DArTag SNPs, PCA followed by discriminant analysis^c^	55	100.0	11	100.0
GBS-generated SNPs, cluster analysis^d^	−	−	8	72.7
DArTag SNPs, cluster analysis^d^	−	−	3	27.3

^a^ Distinctness based on at least one least-significance difference at *P* < 0.01 in analyses for 10 traits.

^b^ Polymorphic markers subjected to a principal components analysis followed by an analysis of variance performed on population scores of individual PC axes; distinctness based on at least one least-significance difference (LSD) at *P* < 0.01.

^c^ Distinctness based on the correct classification of all bulked DNA samples in a discriminant analysis performed on population principal component scores (using one sample per population for validation).

^d^ Distinctness based on the unequivocal and exclusive classification of all bulked DNA samples of a population in the same group with a bootstrap-based probability ≥ 95% at the node of the relevant group.

The statistical criterion based on discriminant analysis performed on population scores of up to five PC axes achieved a complete distinctness of any population from any other (**Table 3**) by several configurations of each marker type. A few configurations (such as those selected for subsequent AMOVA and cluster analyses) achieved a complete distinctness even by using data of one PC axis. DArTag markers tended to be more effective than GBS markers for population distinctness: considering the 100 discriminant analyses per marker type resulting from the combination of 20 marker configurations by one to five possible PC axes used for the analysis, a complete population distinctness was obtained in 71% of the analyses by DArTag markers, and 19% of the analyses by GBS markers.

**Figure 2:**
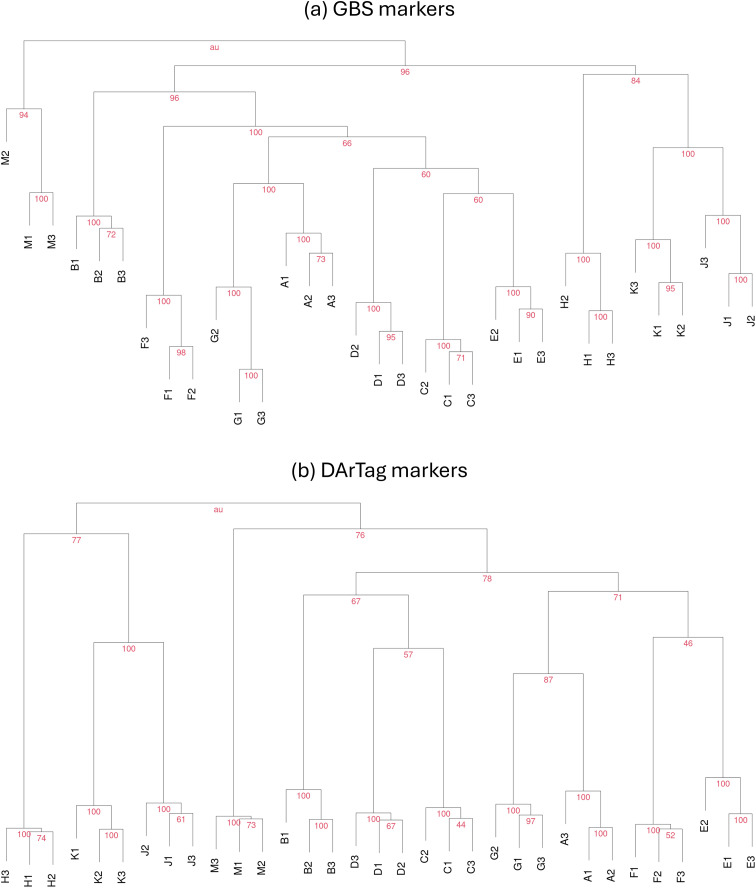
Hierarchical cluster analysis with percent probability values from bootstrap analysis reported at the nodes, performed on Euclidean distances based on allele frequencies of 33 bulked DNA samples relative to 11 alfalfa populations (coded by letters) and three samples per population (indicated by numbers) for **(a)** 10,268 GBS-generated SNP markers, and **(b)** 1569 DArTag SNP markers.

The AMOVA-based statistical criterion did not show any statistical difference at *P* < 0.05 in the paired population comparison. The criterion based on the unequivocal and exclusive classification of all bulked DNA samples of a population in a cluster analysis according to 95% probability of classification in a bootstrap analysis (as reported at the nodes of the groups in **Figure 2**) failed to achieve a complete distinctness of the populations using either marker type (**Table 3**). Three populations out of 11 were not completely distinct according to GBS markers (populations ‘C’, ‘D’, and ‘E’); eight populations were not completely distinct according to DArTag markers (populations ‘A’, ‘B’, ‘C’, ‘D’, ‘E’, ‘F’, ‘G’, and ‘M’) (**Figure 2**). The populations ‘H’, ‘J’ and ‘K’, all derived from selection of previously selfed parents (**Table 1**), were the only ones to be completely distinct according to both marker types (**Figure 2**). The only population that originated from a partly different genetic base, namely ‘M’, was particularly well separated in the cluster analysis performed on GBS markers (**Figure 2**).

The results of Mantel’s test indicated a very high correlation for Euclidean distances of the populations between GBS and DArTag markers (*r* = 0.94; P < 0.001), and no correlation of Euclidean distances based on statistically significant morphophysiological traits with distances based on GBS (*r* = −0.02) or DArTag markers (*r* = −0.05).

## Discussion

Our assessment of population differences for morphophysiological traits using data of one crop cycle instead of two cycles (as prescribed by UPOV) was a limitation that may have had some bearing on our comparison of molecular *versus* morphophysiological distinctness. Data from two crop cycles would imply a larger experiment error in cultivar comparisons for quantitative traits, namely, the cultivar × crop cycle interaction, when the combined over-years distinctness analysis shows significance for this interaction (UPOV, 2022). In that case, the larger error term would have resulted in even less statistically significant population differences than those currently detected. Hence, the reported extent of phenotypic cultivar distinctness may have overestimated what would be obtained in a standard two-cycle DUS trial.

This study not only confirmed the advantage of molecular distinctness over morphophysiological one that was manifest in earlier studies on alfalfa (Annicchiarico et al., 2016b, 2025) but highlighted the extreme advantage of marker-based distinctness in a context of special interest for plant breeding programs, namely, the progressive registration of more varieties selected from the same genetic base. An advantage of molecular distinctness based on GBS markers and the PCA + ANOVA criterion over morphophysiological distinctness in terms of increased number of statistically significant paired comparisons emerged in the earlier studies. The increase was (a) from 43 to 52 out of 55 total comparisons, for 11 Italian historical ecotypes in Annicchiarico et al. (2016b) (who adopted the less conservative value of *P* < 0.05 for all paired comparisons); (b) from 114 to 142 out of 153 total comparisons, for the 18 most grown Italian commercial varieties in Annicchiarico et al. (2025). Here the increase was more drastic, namely, from 20 to 55 statistically significant paired comparisons out of 55. Likewise, the number of completely distinct cultivars, which passed from three based on morphophysiological distinctness to nine or 11 based on GBS or DArTag markers, respectively, in Annicchiarico et al. (2025), here passed from no distinct population to all distinct populations based on GBS or DArTag markers. The absence of variation for proportion of cream, white or yellow flowers was expected, since these colors are usually absent in conventional, mowing-type alfalfa. The current inability to identify even a single population completely distinct morphophysiologically from the others based on the other traits could be attributed to the greater similarity for autumn dormancy, growth habit and plant vigor of the current populations (all selected from the same or a similar genetic base) compared with Italian commercial varieties (Annicchiarico et al., 2025). However, the currently much higher rate of complete molecular distinctness (100% for both marker types) relative to Italian commercial varieties (50% for GBS and 61% for DArTag markers) was unexpected. A possible reason for this result could be the lower within-population genetic variation of most of the current candidate varieties due to their small number of parent genotypes (≤ 15 for 10 populations out of 11: **Table 1**) in comparison with commercial varieties that were mostly selected some decades ago. Details on the number of parents featuring those commercial varieties are not available because of the impossibility to disclose the variety names, but older varieties tend to include a higher number of parent genotypes than recent varieties as a consequence of historically greater reliance on mass selection (Annicchiarico et al., 2016a). An indirect confirmation of lower within-population variation in the current selections was provided by the average variation of the three bulked DNA samples per population acting as replicates in the ANOVA performed of the first two PC axes (**Figure 1**), which was visually smaller than that reported for three bulked DNA samples per commercial variety in Supplementary Figure 1 of Annicchiarico et al. (2025) (for samples issued from identical genotyping procedures). Somewhat lower genetic variation between parent genotypes may partly account for the greater molecular distinctness of the populations ‘H’, ‘J’ and ‘K’ in the cluster analysis (**Figure 2**). These populations derived from selection performed on selfed parent genotypes, and selfing is known to reduce the within-genotype allelic variation contributing to the within-population variation of synthetic varieties.

Our results have important practical implications on the registration of new varieties for an actual breeding program such as ours. Since we already registered the variety ‘Intensa’ (coded as ‘M’), only four of the other 10 selections could be accepted for registration on the ground of sufficient morphophysiological distinctness from this variety. In contrast, all of them could be accepted when considering the molecular distinctness. Remarkably, the absence of morphophysiological distinctness for six selections occurred even if their genetic base was partly different from that used for ‘Intensa’ (**Table 1**). Fortunately, one of the four selections phenologically distinct from ‘Intensa’ was the one coded as ‘C’ (**Supplementary Table 2**), which was the top-yielding selection and out-yielded ‘Intensa’ (*P* < 0.05) in an evaluation trial. This selection is currently under official evaluation for registration in Italy.

Our study contributed to generating information of methodological interest on statistical methods and marker types for alfalfa molecular distinctness. The methods based on PCA followed by ANOVA or discriminant analysis allowed for a complete distinctness of the populations for both marker types. Using ANOVA instead of discriminant analysis could be preferable based on its advantage displayed in two earlier studies (Annicchiarico et al., 2016b, 2025). Both here and in Annicchiarico et al. (2025), the criterion based on cluster analysis was less valuable than that based on PCA + ANOVA, while the criterion based on AMOVA failed to detect any statistical difference between populations. As discussed in Annicchiarico et al. (2025), the ability of AMOVA to reveal statistically distinct populations is severely limited by a low number of bulked DNA samples per cultivar acting as replicates in the analysis, and its results may change markedly already by using four replicates instead of three as in the current study.

GBS and DArTag markers exhibited similar ability to distinguish populations when using PCA followed by ANOVA or discriminant analysis, albeit with somewhat greater efficiency of DArTag markers in the discriminant analysis (as indicated by a higher number of marker configurations that achieved 100% population distinctness). In contrast, GBS markers showed greater ability to distinguish populations according to the statistical method of lesser interest represented by cluster analysis. DArTag markers had shown a slight advantage over GBS markers when using any statistical method in Annicchiarico et al. (2025). The mostly comparable sensitivity for cultivar distinctness exhibited by DArTag markers relative to GBS ones is remarkable, when considering their much lower number of polymorphic markers (1569 *versus* 10,268 for the two well-performing marker configurations) and the fact than even under the most liberal configuration only 60% of the markers belonging to the DArTag panel showed polymorphism in our material (1799 out of 3000: **Supplementary Table 1**). The modest frequency of polymorphic DArTag markers could be related to their selection from 40 cultivated alfalfa parents and founders from North American breeding programs (Zhao et al., 2023). The disadvantage of DArTag due to much lower marker number was counterbalanced by a very good marker dispersion across the genome and by better estimation of allele marker frequencies relative to GBS markers due to about 10-fold greater average reading depth per marker (596 *versus* 64 for the two well-performing marker configurations). The DArTag panel could be recommended for alfalfa molecular distinctness also because of the following advantages relative to GBS markers: (a) about two-third lower cost per genotyped cultivar (at least in our studies); (b) an open-access technology; (c) a somewhat simpler SNP calling pipeline, and lower amount of missing data.

The complete lack of correlation between population diversity according to morphophysiological traits and diversity based on GBS or DArTag markers was affected by the limited morphophysiological diversity featuring the alfalfa materials. However, Arcia-Ruiz et al. (2025), building on the genomic framework established by Pégard et al. (2023), revealed a weak correlation between phenotypic and molecular diversity (Mantel’s *r* = 0.17) even for an international collection of 395 cultivated alfalfa accessions. A modest consistency between marker-based and morphophysiological cultivar diversity emerged in several earlier studies concerning cultivars of alfalfa (Crochemore et al., 1998; Annicchiarico et al., 2016b, 2025) and other perennial legume (Kölliker et al., 2001; Greene et al., 2004; Dias et al., 2008) or perennial grass species (Roldán-Ruiz et al., 2001; Sun et al., 2015; Roberts et al., 2026), with the exception of the fairly high correlation reported in one alfalfa study by Julier et al. (2018). Particularly for alfalfa, the modest correlation between morphophysiological and molecular diversity for sets of material ranging from national cultivars (Annicchiarico et al., 2025) to a vast, international germplasm collection (Arcia-Ruiz et al., 2025) does not encourage the use of molecular diversity as a proxy for morphophysiological diversity in the management of reference collections aimed to identify a small set of control cultivars with greatest expected morphophysiological similarity with that of the candidate variety (UPOV, 2020). This conclusion contrasts with findings for major inbred crops, for which morphophysiological and molecular diversity mostly showed a moderate consistency (Jones et al., 2013; Achard et al., 2020).

Our assessment of molecular distinctness was based on the overall diversity of the populations for a large number of DNA markers that are mostly selectively neutral and not linked to any morphophysiological trait. This approach has received much attention also for inbred crops (Jones et al., 2013; Jamali et al., 2019, 2020; Owen et al., 2019; Achard et al., 2020). An alternative approach would be to consider only the markers linked to morphophysiological traits, as devised by Roberts et al. (2026) in a study on wheat and perennial ryegrass and in other studies on outbred (Jo et al., 2022) or inbred crops (Jones and Mackay, 2015). This latter approach would suffer two limitations for alfalfa. The first is the scarcity of valuable morphophysiological traits for alfalfa, as highlighted in Annicchiarico et al. (2025) for a morphologically wider set of cultivars, which the use of markers as a proxy for the ordinary morphophysiological traits could not overcome. The second is the prerequisite of high predictive ability for the markers used as a proxy for a given morphophysiological trait, which is difficult to satisfy for alfalfa based on the available information. Even the trait with highest genome-enabled predictive ability, namely autumn plant height, displayed a prediction accuracy in the range of 0.50-0.65 (Jia et al., 2018; Wang et al., 2024). Prediction abilities were in the range of 0.30-0.50 for onset of flowering (Jia et al., 2018; Pégard et al., 2023; Nazzicari et al., 2024); mostly in the range of 0.30-0.60 for plant height (Andrade et al., 2022; Pégard et al., 2023; Wang et al., 2024); and around 0.30 for leaflet size (Nazzicari et al., 2024). Actually, Roberts et al. (2026) aimed to exploit genomic predictions of DUS traits to reduce the number of control varieties used for the field-based assessment of morphophysiological distinctness of a candidate variety and, later on, to strengthen the indications of the morphophysiological assessment. Following Gilliland et al. (2020), our proposed use of molecular diversity is to provide an additional criterion to verify the distinctness of candidate varieties in case of lack of morphophysiological distinctness.

## Conclusion

Variety registration has been designed as a mechanism to reward plant breeding investment and to support farmers and the broader agri-food sector by ensuring that new varieties represent genuine improvements over existing ones. The distinctness requirement, historically based necessarily on morphophysiological characteristics, has been part of this process by granting breeders an exclusive right over identifiable varieties. For alfalfa, the exclusion from registration of agronomically valuable varieties and the increasingly difficult protection of Plant Breeding Rights that derive from reliance on morphophysiological distinctness undermine the very aims of the variety registration system and represent the strongest possible argument for reforming the distinctness assessment by also including the molecular distinctness. This study provides additional evidence for the considerable advantage for alfalfa breeding programs represented by molecular distinctness as a support to ordinary DUS, thereby confirming the interest and urgency of a reform of the procedures for DUS assessment for this and other major perennial forages (Gilliland et al., 2020). The genetic variation provided by molecular markers was currently able to distinguish all populations despite their genetic similarity and the fact that Po Valley germplasm accounts for a narrow slice of the global genetic diversity observed in cultivated alfalfa (Pégard et al., 2023). Molecular distinctness could conveniently rely on the current DArTag panel. A novel DArTag panel with a specific focus on European germplasm may be envisaged in the future in case the current panel was not sufficiently satisfactory for the challenging discrimination of cultivars belonging to the entire European reference collection.

As argued in many reports (Wang et al., 2019; Gilliland et al., 2020; Jamali et al., 2020; Yang et al., 2021), molecular distinctness, besides being more sensitive, offers additional important advantages relative to morphophysiological distinctness in terms of test duration (some months *versus* three years), cost, and reliability of the assessment (with respect to genotype × environment interactions affecting quantitative traits). For example, the molecular assessment could be verified at the very beginning of the variety registration process, providing feedback sufficiently timely to prevent the onset of the VCU assessment (or at least its prolongation beyond the first year) in the absence of acceptable variety distinctness. As anticipated, the risk of variety plagiarism by a drastic change of allele frequencies becomes irrelevant in perennial forage crops when the assessment of molecular distinctness is based on a very large number of markers.

Other important potential advantages of molecular distinctness concern its timely, reliable and cost-efficient application in two important contexts, namely, forensic analyses for lawsuits (where the variety protection is practically enforced), and the control of certified seed. With regard to the latter, officers charged of field inspections for certified seed production in Italy repeatedly reported the impossibility to verify the identity of alfalfa varieties under multiplication on the basis of morphophysiological traits (Annicchiarico et al., 2025), while the morphophysiological trait-based control of random samples of certified seed lots suffers of the same time and cost limitations as the DUS assessment. Italian farmers producing dehydrated alfalfa consider high the risk of seed adulteration in alfalfa varieties, owing to the difficult control of certified seed (R. Severi and T. Migiani, pers. comm., 2025). Developing a reliable marker-based system of certified seed control may require, however, additional information on the impact of different environments and generations of seed multiplication on the overall marker variation.

Our study focused on alfalfa, but similar limitations of ordinary DUS and opportunities for molecular distinctness are likely to feature other major perennial forages bred as synthetic varieties. While initially established as a support to ordinary DUS (Gilliland et al., 2020), molecular distinctness may ultimately replace the morphophysiological distinctness and encompass the entire DUS assessment, if markers could reliably be devised also for uniformity and stability assessments. Recent work by Roberts et al. (2025) showed that molecular markers could also be used for assessing the uniformity requirement of candidate forage crop varieties.

## Data Availability

Molecular datasets used in this study are available in the online repository: https://doi.org/10.6084/m9.figshare.31323901.

## References

[B1] Achard F. Butruille M. Madjarac S. Nelson P. T. Duesing J. Laffont J.-L. . (2020). Single nucleotide polymorphisms facilitate distinctness-uniformity-stability testing of soybean cultivars for plant variety protection. Crop Sci. 60, 2280–2303. doi: 10.1002/csc2.20201, PMID: 41925066

[B2] Andrade M. H. M. L. Acharya J. P. Benevenuto J. de Bem Oliveira I. Lopez Y. Munoz P. . (2022). Genomic prediction for canopy height and dry matter yield in alfalfa using family bulks. Plant Genome 15, e20235. doi: 10.1002/tpg2.20235, PMID: 35818699 PMC12807079

[B3] Annicchiarico P. (2021). Breeding gain from exploitation of regional adaptation: an alfalfa case study. Crop Sci. 61, 2254–2270. doi: 10.1002/csc2.20423, PMID: 41925066

[B4] Annicchiarico P. Barrett B. Brummer E. C. Julier B. Marshall A. H. (2015). Achievements and challenges in improving temperate perennial forage legumes. Crit. Rev. Plant Sci. 34, 327–380. doi: 10.1080/07352689.2014.898462, PMID: 41909888

[B5] Annicchiarico P. Boller B. Brummer E. C. Reheul D. (2016a). “ Improving the focus of forage breeding research,” in Breeding in a world of scarcity. Eds. Baert J. Reheul D. Roldan I. ( Springer, Dordrecht, The Netherlands), 251–269.

[B6] Annicchiarico P. Franguelli N. Ferrari B. Campanella G. Gualanduzzi S. Crosta M. . (2025). Molecular markers enhance substantially the distinctness of alfalfa varieties for registration and protection. Plant Genome 18, e20556. doi: 10.1002/tpg2.20556, PMID: 39906928 PMC11795343

[B7] Annicchiarico P. Nazzicari N. Ananta A. Carelli M. Wei Y. Brummer E. C. (2016b). Assessment of cultivar distinctness in alfalfa: a comparison of genotyping-by-sequencing, SSR marker and morphophysiological observations. Plant Genome 9, plantgenome2015.10.0105.2. doi: 10.3835/plantgenome2015.10.0105, PMID: 27898838

[B8] Annicchiarico P. Pecetti L. (2021). Comparison among nine alfalfa breeding schemes based on actual biomass yield gains. Crop Sci. 61, 2355–2370. doi: 10.1002/csc2.20464, PMID: 41925066

[B9] Arcia-Ruiz I. Pégard M. Surault F. Živanov D. Milić D. Karagić Đ. . (2025). Phenotypic and genomic insights into alfalfa diversity: identifying critical loci for enhanced resilience. Plant Genome 18, e70155. doi: 10.1002/tpg2.70155, PMID: 41261851 PMC12631055

[B10] Busbice T. H. (1969). Inbreeding in synthetic varieties. Crop Sci. 9, 601–604. doi: 10.2135/cropsci1969.0011183X000900050026x

[B11] Byrne S. Czaban A. Studer B. Panitz F. Bendixen C. Asp T. (2013). Genome wide allele frequency fingerprints (GWAFFs) of populations via genotyping by sequencing. PloS One 8, e57438. doi: 10.1371/journal.pone.0057438, PMID: 23469194 PMC3587605

[B12] Crochemore M.-L. Huyghe C. Écalle C. Julier B. (1998). Structuration of alfalfa genetic diversity using agronomic and morphological characteristics. Relationship with RAPD markers. Agronomie 18, 79–94. doi: 10.1051/agro:19980106

[B13] Dias P. M. B. Julier B. Sampoux J.-P. Barre P. Dall’Agnol M. (2008). Genetic diversity in red clover (*Trifolium pratense* L.) revealed by morphological and microsatellite (SSR) markers. Euphytica 160, 189–205. doi: 10.1007/s10681-007-9534-z, PMID: 41933263

[B14] Diversity Arrays Technology (2026). Targeted genotyping services ( Diversity Arrays Technology). Available online at: https://www.diversityarrays.com/services/targeted-genotying/.

[B15] Elshire R. J. Glaubitz J. C. Sun Q. Poland J. A. Kawamoto K. Buckler E. S. . (2011). A robust, simple genotyping-by-sequencing (GBS) approach for high diversity species. PloS One 6, e19379. doi: 10.1371/journal.pone.0019379, PMID: 21573248 PMC3087801

[B16] EUPVP (2025). Common catalogue information system ( EU Plant Variety Portal). Available online at: https://ec.europa.eu/food/plant-variety-portal/.

[B17] Gilliland T. J. Annicchiarico P. Julier B. Ghesquière M. (2020). A proposal for enhanced EU herbage VCU and DUS testing procedures. Grass. Forage. Sci. 75, 227–241. doi: 10.1111/gfs.12492, PMID: 41940437

[B18] Gilliland T. J. Gensollen V. (2010). “ Review of the protocols used for assessment of DUS and VCU in Europe – Perspectives,” in Sustainable use of genetic diversity in forage and turf breeding. Ed. Huyghe C. ( Springer, Dordrecht, The Netherlands), 261–275. doi: 10.1007/978-90-481-8706-5_37, PMID:

[B19] Greene S. L. Gritsenko M. Vandemark G. (2004). Relating morphologic and RAPD marker variation to collection site environment in wild populations of red clover (*Trifolium pratense* L.). Genet. Resour. Crop Evol. 51, 643–653. doi: 10.1023/B:GRES.0000024655.48989.ab, PMID: 38124636

[B20] Herrmann D. Flajoulot S. Barre P. Huyghe C. Ronfort J. Julier B. (2018). Comparison of morphological traits and molecular markers to analyse diversity and structure of alfalfa (*Medicago sativa* L.) cultivars. Genet. Resour. Crop Evol. 65, 527–540. doi: 10.1007/s10722-017-0551-z, PMID: 41933263

[B21] Jamali S. H. Cockram J. Hickey L. T. (2019). Insights into deployment of DNA markers in plant variety protection and registration. Theor. Appl. Genet. 132, 1911–1929. doi: 10.1007/s00122-019-03348-7, PMID: 31049631

[B22] Jamali S. H. Cockram J. Hickey L. T. (2020). Is plant variety registration keeping pace with speed breeding techniques? Euphytica 216, 131. doi: 10.1007/s10681-020-02666-y, PMID: 41933263

[B23] Jia C. Zhao F. Wang X. Han J. Zhao H. Liu G. . (2018). Genomic prediction for 25 agronomic and quality traits in alfalfa (*Medicago sativa*). Front. Plant Sci. 9. doi: 10.3389/fpls.2018.01220, PMID: 30177947 PMC6109793

[B24] Jo J. Kang M. Y. Kim K. S. Youk H. R. Shim E. J. Kim H. . (2022). Genome-wide analysis-based single nucleotide polymorphism marker sets to identify diverse genotypes in cabbage cultivars (*Brassica oleracea* var. capitata). Sci. Rep. 12, 20030. doi: 10.1038/s41598-022-24477-y, PMID: 36414667 PMC9681867

[B25] Jones H. Mackay I. (2015). Implications of using genomic prediction within a high-density SNP dataset to predict DUS traits in barley. Theor. Appl. Genet. 128, 2461–2470. doi: 10.1007/s00122-015-2601-2, PMID: 26350495

[B26] Jones H. Norris C. Smith D. Cockram J. Lee D. O’Sullivan D. M. . (2013). Evaluation of the use of high-density SNP genotyping to implement UPOV Model 2 for DUS testing in barley. Theor. Appl. Genet. 126, 901–911. doi: 10.1007/s00122-012-2024-2, PMID: 23232576

[B27] Julier B. Barre P. Lambroni P. Delaunay S. Thomasset M. Lafaillette F. . (2018). Use of GBS markers to distinguish among lucerne varieties, with comparison to morphological traits. Mol. Breed. 38, 133. doi: 10.1007/s11032-018-0891-1, PMID: 41933263

[B28] Khan H. A. Arif I. A. Bahkali A. H. Farhan A. H. A. Homaidan A. A. A. (2008). Bayesian, maximum parsimony and UPGMA models for inferring the phylogenies of antelopes using mitochondrial markers. Evol. Bioinform. 4, EBO–S934. doi: 10.4137/EBO.S934, PMID: 19204824 PMC2614192

[B29] Kölliker R. Jones E. S. Jahufer M. Z. Z. Forster J. W. (2001). Bulked AFLP analysis for the assessment of genetic diversity in white clover (*Trifolium repens* L.). Euphytica 121, 305–315. doi: 10.1023/A:1012048103585, PMID: 41886696

[B30] Legendre P. Legendre L. (2012). Numerical ecology. 3rd ed (Amsterdam, The Netherlands: Elsevier).

[B31] Li X. Brummer E. C. (2012). Applied genetics and genomics in alfalfa breeding. Agronomy 2, 40–61. doi: 10.3390/agronomy2010040, PMID: 41725453

[B32] Long R. Zhang F. Zhang Z. Li M. Chen L. Wang X. . (2022). Genome assembly of alfalfa cultivar Zhongmu-4 and identification of SNPs associated with agronomic traits. GPB 20, 14–28. doi: 10.1016/j.gpb.2022.01.002, PMID: 35033678 PMC9510860

[B33] Malmberg M. M. Suraweera D. D. Baillie R. C. Smith K. F. Cogan N. O. I. (2025). Genomic resources for Australian alfalfa (*Medicago sativa* L.) genomics: reformatted reference genome, annotated variants, gene presence-absence and diversity analysis from genome re-sequencing. BMC Plant Biol. 26, 102. doi: 10.1186/s12870-025-07941-5, PMID: 41402760 PMC12822192

[B34] Nazzicari N. Franguelli N. Ferrari B. Pecetti L. Annicchiarico P. (2024). The effect of genome parametrization and SNP marker subsetting on genomic selection in autotetraploid alfalfa. Genes 15, 449. doi: 10.3390/genes15040449, PMID: 38674384 PMC11050091

[B35] Nei M. (1972). Genetic distance between populations. Am. Nat. 106, 283–292. doi: 10.1086/282771

[B36] OECD (2025). Agriculture and fisheries ( Organisation for Economic Co-operation and Development). Available online at: https://www.oecd.org/agriculture/seeds/varieties/.

[B37] Owen H. Pearson K. Roberts A. M. I. Reid A. Russell J. (2019). Single nucleotide polymorphism assay to distinguish barley (*Hordeum vulgare* L.) varieties in support of seed certification. Genet. Resour. Crop Evol. 66, 1243–1256. doi: 10.1007/s10722-019-00785-7, PMID: 41933263

[B38] Pégard M. Barre P. Delaunay S. Surault F. Karagić D. Milić D. . (2023). Genome-wide genotyping data renew knowledge on genetic diversity of a worldwide alfalfa collection and give insights on genetic control of phenology traits. Front. Plant Sci. 14. doi: 10.3389/fpls.2023.1196134, PMID: 37476178 PMC10354441

[B39] Pembleton L. W. Cogan N. O. I. Forster J. W. (2013). StAMPP: an R package for calculation of genetic differentiation and structure of mixed-ploidy level populations. Mol. Ecol. Resour. 13, 946–952. doi: 10.1111/1755-0998.12129, PMID: 23738873

[B40] Pohelman J. M. Sleper D. A. (1995). Breeding field crops. 4th ed (Ames, IA: Iowa State University Press).

[B41] Pupilli F. Businelli S. Paolocci F. Scotti C. Damiani F. Arcioni S. (1996). Extent of RFLP variability in tetraploid populations of alfalfa, *Medicago sativa*. Plant Breed. 115, 106–112. doi: 10.1111/j.1439-0523.1996.tb00883.x, PMID: 41940437

[B42] Roberts A. M. I. Barre P. Delaunay S. Julier B. Byrne S. Milbourne D. (2025). Assessing varietal heterogeneity in pooled samples of cross-pollinated crops using genetic markers. Grass. Forage. Sci. 80, e70009. doi: 10.1111/gfs.70009, PMID: 41940437

[B43] Roberts A. M. I. Neugebauer K. Ewaoluwagbemiga E. O. Milbourne D. Byrne S. Cockram J. . (2026). Integrating genomic prediction into crop DUS testing: new frameworks for Reference Collection management and Distinctness assessment. Theor. Appl. Genet. 139, 93. doi: 10.1007/s00122-026-05198-6, PMID: 41817728 PMC12982246

[B44] Roldán-Ruiz I. Van Euwijk F. A. Gilliland T. J. Dubreuil P. Dillmann C. Lallemand J. . (2001). A comparative study of molecular and morphological methods of describing relationships between perennial ryegrass (*Lolium perenne* L.) varieties. Theor. Appl. Genet. 103, 1138–1150. doi: 10.1007/s001220100571, PMID: 41933263

[B45] Shimodaira H. (2002). An approximately unbiased test of phylogenetic tree selection. Syst. Biol. 51, 492–508. doi: 10.1080/10635150290069913, PMID: 12079646

[B46] Sun X. Xie Y. Bi Y. Liu J. Amombo E. Hu T. . (2015). Comparative study of diversity based on heat tolerant-related morpho-physiological traits and molecular markers in tall fescue accessions. Sci. Rep. 5, 18213. doi: 10.1038/srep18213, PMID: 26666506 PMC4678371

[B47] Suzuki R. Terada Y. Shimodaira H. (2019). “ pvclust: hierarchical clustering with P-values via multiscale bootstrap resampling,” in R package version 2.2-0. Available online at: https://CRAN.R-project.org/package=pvclust. (Accessed February 10, 2026).

[B48] UPOV (1990). Harmonization of states of expression and notes of characteristics, Document TC/26/4 Rev. (Geneva, Switzerland: UPOV).

[B49] UPOV (1991). International convention for the protection of new varieties of plants, Publication no. 221(E) (Geneva, Switzerland: UPOV).

[B50] UPOV (2004). Molecular techniques, document CAJ/50/4 (Geneva, Switzerland: UPOV).

[B51] UPOV (2005). Lucerne, document TG/6/5 (Geneva, Switzerland: UPOV).

[B52] UPOV (2015). Examining distinctness, document TGP/9 (Geneva, Switzerland: UPOV).

[B53] UPOV (2020). *Guidance on the use of biochemical and molecular markers in the examination of Distinctness, Uniformity and Stability (DUS)*, *Document TGP/15* (Geneva, Switzerland: UPOV).

[B54] UPOV (2022). Trial design and techniques used in the examination of Distinctness, Uniformity and Stability, Document TGP/8 (Geneva, Switzerland: UPOV).

[B55] Van Eeuwijk F. Law J. (2004). Statistical aspects of essential derivation, with illustrations based on lettuce and barley. Euphytica 137, 129–137. doi: 10.1023/B:EUPH.0000040510.31827.ae, PMID: 38124636

[B56] Van Wijk A. Louwaars N. (2014). Framework for the introduction of Plant Breeder’s Rights: Guidance for practical implementation (Roelofarendsveen, The Netherlands: Naktuinbouw).

[B57] Vaz C. Nascimento M. Carriço J. A. Rocher T. Francisco A. P. (2021). Distance-based phylogenetic inference from typing data: a unifying view. Brief Bioinform. 22, bbaa147. doi: 10.1093/bib/bbaa147, PMID: 32734294

[B58] Wang H. Bai Y. Biligetu B. (2024). Effects of SNP marker density and training population size on prediction accuracy in alfalfa (*Medicago sativa* L.) genomic selection. Plant Genome 17, e20431. doi: 10.1002/tpg2.20431, PMID: 38263612 PMC12807208

[B59] Wang F. G. Tian H. L. Yi H. M. Zhao H. Huo Y. X. Kuang M. . (2019). Principle and strategy of DNA fingerprint identification of plant variety. Mol. Plant Breed. 10, 81–92. doi: 10.5376/mpb.2019.10.0011

[B60] Wang J. Wei X. Guo C. Xu C. Zhao Y. Pu X. . (2025). Simple sequence repeat-based genetic diversity analysis of alfalfa varieties. Int. J. Mol. Sci. 26, 5246. doi: 10.3390/ijms26115246, PMID: 40508055 PMC12154066

[B61] Wilkins P. W. Humphreys M. O. (2003). Progress in breeding perennial forage grasses for temperate agriculture. J. Agric. Sci. 140, 129–150. doi: 10.1017/S0021859603003058, PMID: 41292463

[B62] Yang C. J. Russell J. Ramsay L. Thomas W. Powell W. Mackay I. (2021). Overcoming barriers to the registration of new plant varieties under the DUS system. Commun. Biol. 4, 302. doi: 10.1038/s42003-021-01840-9, PMID: 33686157 PMC7940638

[B63] Yu Q. Ling Y. Xiong Y. Zhao W. Xiong Y. Dong Z. . (2022). RAD-seq as an effective strategy for heterogenous variety identification in plants – a case study in Italian ryegrass (*Lolium multiflorum*). BMC Plant Biol. 22, 231. doi: 10.1186/s12870-022-03617-6, PMID: 35513782 PMC9069751

[B64] Zhao D. Mejia-Guerra K. M. Mollinari M. Samac D. Irish B. Heller-Uszynska K. . (2023). A public mid-density genotyping platform for alfalfa (*Medicago sativa* L.). Genet. Resour. 4, 55–63. doi: 10.46265/genresj.EMOR6509

