# Supplementary material for: Implications of morphological and molecular distinctness on the registration of alfalfa candidate varieties issued by a breeding program: a case study
Source: Front Plant Sci. 2026 Apr 14;17:1809996. doi: 10.3389/fpls.2026.1809996 (PMC13121342; doi:10.3389/fpls.2026.1809996)
Supplement: Supplementary file 1 [file Table1.doc]

**Supplementary Table S1.** Number of polymorphic GBS-generated and DArTag SNP markers as a function of different thresholds of maximum missing genotypes per marker (mpm) and minimum reads per marker (rm).

|  | **rm, GBS-generated SNPs** | | | |  | **rm, DArTag SNPs** | | | |
| --- | --- | --- | --- | --- | --- | --- | --- | --- | --- |
| **mpm** | **10** | **20** | **30** | **40** |  | **10** | **20** | **30** | **40** |
| 0.01 | 12,966 | 6341 | 3224 | 1471 |  | 1344 | 1306 | 1264 | 1210 |
| 0.05 | 18,156 | 10,359 | 6196 | 3436 |  | 1533 | 1504 | 1471 | 1441 |
| 0.10 | 23,806 | 14,684 | 10,268 | 6932 |  | 1624 | 1601 | 1569 | 1535 |
| 0.20 | 29,343 | 18,781 | 13,700 | 10,182 |  | 1729 | 1698 | 1664 | 1640 |
| 0.30 | 33,560 | 21,824 | 16,256 | 12,509 |  | 1799 | 1770 | 1744 | 1713 |
