# Supplementary material for: Implications of morphological and molecular distinctness on the registration of alfalfa candidate varieties issued by a breeding program: a case study
Source: Front Plant Sci. 2026 Apr 14;17:1809996. doi: 10.3389/fpls.2026.1809996 (PMC13121342; doi:10.3389/fpls.2026.1809996)
Supplement: Supplementary file 2 [file Table2.docx]

| Populations | B | C | D | E | F | G | H | J | K | M |
| --- | --- | --- | --- | --- | --- | --- | --- | --- | --- | --- |
| A | - | - | VF | - | - | - | - | - | - | - |
| B |  | - | OF | VF | - | - | - | VF | OF | OF; VF |
| C |  |  | LL; VF | DF | - | - | - | - | - | LL |
| D |  |  |  | DF; VF | - | VF | VF | VF | VF | VF |
| E |  |  |  |  | VF | - | - | DF | - | DF |
| F |  |  |  |  |  | - | - | - | OF | - |
| G |  |  |  |  |  |  | - | - | OF | - |
| H |  |  |  |  |  |  |  | - | - | - |
| J |  |  |  |  |  |  |  |  | - | - |
| K |  |  |  |  |  |  |  |  |  | - |

**Supplementary Table S2.** **Morphophysiological traits showing difference in pairwise comparisons between 11 alfalfa populations according to Fisher’s least significant difference (*P* < 0.01). Trait abbreviations: OF, onset of flowering; DF, proportion of plants with very dark flower; VF, proportion of plants with variegated flower; LL, length of central leaflet. See Table 1 for description of populations.**
